# Using multiple case studies of health and justice services to inform the development of a new complex intervention for prison-leavers with common mental health problems (Engager)

**DOI:** 10.1186/s40352-021-00131-z

**Published:** 2021-02-17

**Authors:** Charlotte Lennox, Rachel Stevenson, Christabel Owens, Richard Byng, Sarah L. Brand, Mike Maguire, Graham Durcan, Caroline Stevenson, Jenny Shaw, Cath Quinn

**Affiliations:** 1 Division of Psychology and Mental Health, The University of Manchester, 2.315 Jean McFarlane Building, Oxford Road, Manchester, M13 9PL UK; 2 University of Exeter Medical School, St Luke’s Campus, Exeter, EX1 2LU UK; 3 Community and Primary Care Research Group, University of Plymouth, Drake Circus, Plymouth, Devon, PL4 8AA UK; 4 Centre for Criminology, University of South Wales, Pontypridd, CF37 1DL Wales; 5 Centre for Mental Health, South Bank Technopark, 90 London Rd, London, SE1 6LD UK

**Keywords:** Case study design, Health services, Justice services, Common mental health, Interagency working, Collaboration, Engagement

## Abstract

**Background:**

People in the criminal justice system have complex needs but often do not make use of services outside of prison, in many cases due to poorly joined up working between health and criminal justice services. The ‘Engager’ programme aimed to develop a complex collaborative care intervention for people leaving prison with common mental health problems that could support their transition into the community and facilitate joined up working between health, justice and social services. To augment our core intervention theory, we wanted to learn from innovative and forward-thinking services providing interagency support and/or treatment for people experiencing common mental health problems within the criminal justice system. We wanted to identify key elements of interagency practice to understand what was and was not effective in engaging people, maintaining their contact and improving mental health and other aspects of their lives.

**Method:**

We used a multiple case study design with a focused ethnographic approach in four study sites. Data came from three sources (documents, field notes and semi-structured interviews) underwent a framework analysis.

**Results:**

We identified seven main themes, namely: collaboration, client engagement, client motivation, supervision, therapeutic approach, peers and preparations for ending. Engaging and motivating clients was dependent on the relationship built with the professional. This relationship was developed through building trust and rapport, which required time and respectful, open and honest communication. Professionals were often unable to build this relationship effectively if they did not work in effective interagency collaborations, particularly those which included shared practices and were supported by effective supervision.

**Conclusions:**

The multiple case study design contributed insights as to how health and justice services work together. The main themes identified are well known factors in health and justice co-working. However, the novel insights were gleaned examining interdependence and interactions in complex, multifactorial phenomena and practice, in particular the importance of shared practice and supervision models. The approach of selecting a small number of cases representing identified knowledge gaps contributed a valuable addition to the program theory and delivery for an innovative complex intervention.

## Introduction

Individuals coming out of prison experience a combination of complex mental health problems, social exclusion and resettlement difficulties (Hamilton & Belenko, 2016; Mallik-Kane & Visher, 2008; Toi & Mogro-Wilson, 2015; Wallace et al., 2016). While those with substance use disorders and severe mental illness, such as psychosis, are likely to be in contact with specialist services, others with common mental health problems (anxiety, depression, post-traumatic stress disorder) are unlikely to be in regular contact with any service (Byng et al., 2012; Shaw et al., 2017). There is a dearth of robust evidence-based literature to inform the delivery of services for those with common mental health problems. There are, however, isolated pockets of innovative practice that have attempted to overcome some of the challenges faced by both individuals and services. This paper describes the use of case studies of such innovative services to inform the delivery of a complex collaborative care intervention for men with common mental health problems in the UK (‘Engager’), the aim of which was to support their transition from prison to the community.

People in contact with all parts of the Criminal Justice System (CJS), but especially those in prison, have a high prevalence of mental health problems, much higher than that found in the general population (Brooke, Taylor, Gunn, & Maden, 1996; Brugha et al., 2005; Fazel & Danesh, 2002; Harding, Wildgoose, Sheeran, Beckley, & Regan, 2007; Mair & May, 1997; Rekrut-Lapa & Lapa, 2014; Singleton, Meltzer, Gatward, Coid, & Deasy, 1998; Sirdifield, 2012; Stewart, 2008). They also often have co-morbidities, including physical (Ministry of Justice, 2012; Public Health England, 2014) and/or substance misuse problems (Light, Grant, & Hopkins, 2013) and a wide range of personal and social problems. Those in contact with the CJS frequently lead chaotic lives, typically including homelessness, unemployment, and broken relationships with their partners and children (Prison Reform Trust, 2018).

Despite their complex needs, the evidence suggests that those in contact with the CJS often make little use of health services while outside of prison (Harty, Tighe, Leese, Parrott, & Thornicroft, 2003; Lennox et al., 2012). In theory, once released into the community, those with common mental health problems are provided for by mainstream statutory services including general practice, community mental health teams, and Increasing Access to Psychological Therapies (IAPT) services. However, in reality few access these services (Byng et al., 2012).

Given the complexity of needs in this population that separately challenge the CJS and health service, inter-agency collaboration is essential (Lennox, Mason, McDonnell, Shaw, & Senior, 2012). Government publications have recognised the need for improvements in CJS health care (Bradley, 2009; Department of Health, 2009), Identifying a habit of ‘working in silos’ which has resulted in disconnected policies and practices, differing workloads, a lack of information sharing and insufficient opportunity to meet and share ideas (Bradley, 2009). They advocate a joined-up approach to services, with effective interagency working throughout the entire care pathway. However, despite the political drive to improve health and CJS partnership working, there is evidence that services have struggled to implement such policies (House of Commons, 2018; National Audit Office, 2017; Public Health England, 2016) resulting in individuals falling between gaps in services, a lack of clear accountability, and poor continuity of care.

In 2013 the Government published *Transforming Rehabilitation: A strategy for reform* (Ministry of Justice, 2013) which aimed to promote more co-ordinated and ‘innovative’ inter-agency work with prison leavers through the development of supply chains of service providers, including third sector organisations, led mainly by large private sector ‘primes’ (Maguire, 2016). Community Rehabilitation Companies (CRCs) were established to provide a nationwide through the prison gate resettlement service to provide continuous support from custody into the community, for low to medium risk offenders (the National Probation Service (NPS) remained responsible for high risk offenders). However, substantial concerns have been highlighted, including difficult working relationships between the CRCs and NPS, limited partnership working between the CRCs and voluntary organisations and poor through the gate support (National Audit Office, 2016, 2019). As a result, the Government announced the end of the CRC contracts in 2020 – the latest in a long history of failed attempts to create effective ‘joined up’ resettlement services for prisoners.

The aim of the ‘Engager’ programme was to develop and evaluate a complex collaborative care intervention for prisoners with common mental health problems that supports their transition into the community and facilitates joined-up working between health and CJS services (Kirkpatrick et al., 2018). ‘Engager’ was iteratively developed from a number of sources, including a realist review of the scientific and grey literatures (Pearson et al., 2015), a series of focus groups (Owens, Carter, Shenton, Byng, & Quinn, 2018), the lived experience of a group of peer researchers (Taylor, Gill, Gibson, Byng, & Quinn, 2018), and a realist formative process evaluation (Brand et al., 2019) embedded in a pilot trial (Lennox et al., 2017). In designing and planning its delivery, we wanted to try and identify key elements of practice and to understand what was, and was not, effective in helping to engage people, maintain their contact and improve their mental health and general wellbeing. To achieve this, we decided to produce some detailed case studies of innovative and forward-looking services where health and criminal justice partnerships worked well.

**Fig. 1:**
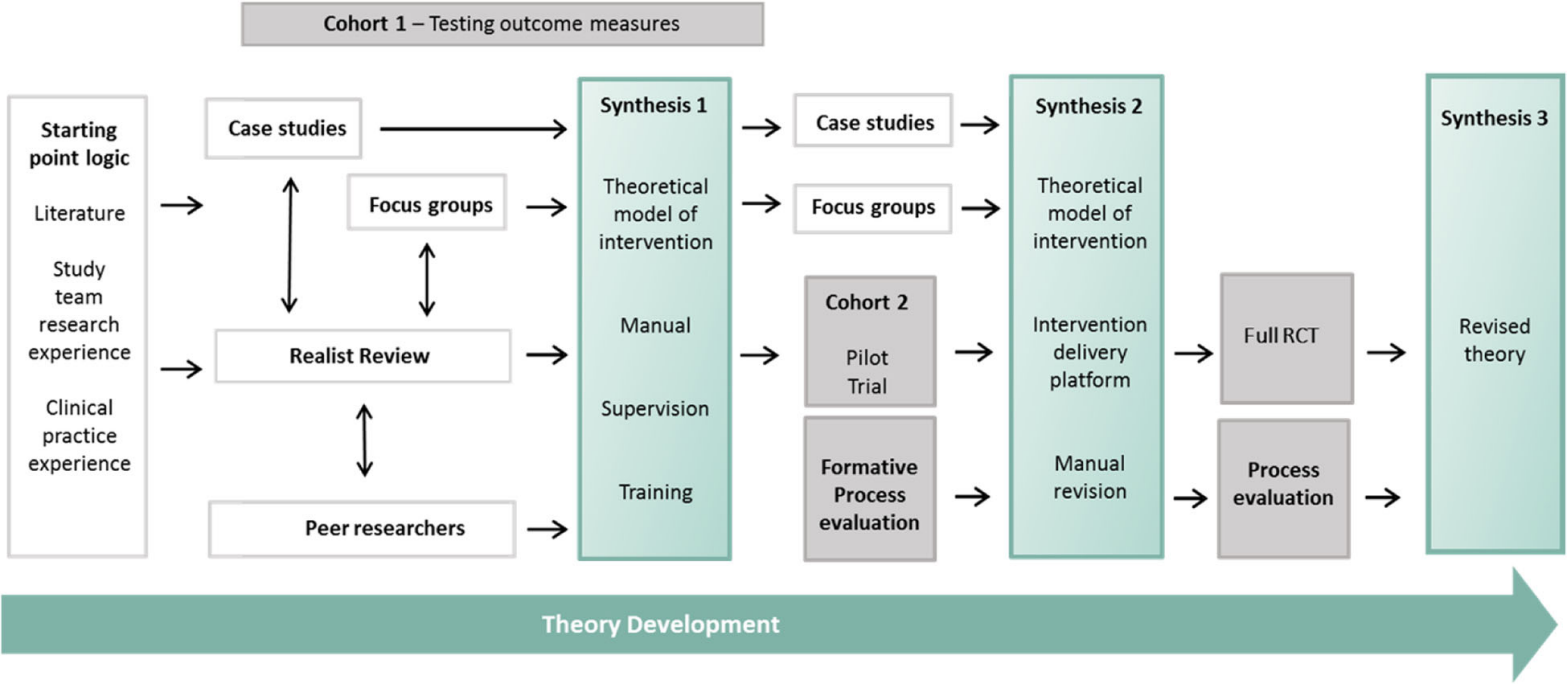
Overview of the ‘Engager’ programme

Figure 1 provides an overview of the ‘Engager’ programme and shows where the case studies occurred in the theory development timeline. Our initial program theory had three important presumptions; 1) ‘Engager’ practitioners are the main intervention resource, and together with the prison leaver produce outcomes; 2) individuals’ problems are diverse, therefore flexibility was prioritised over fixed protocolisation of intervention components; 3) ensuring delivery in challenging contexts would require substantial and well-focussed resource and a comprehensive implementation platform would be required. Therefore, to augment theory based initially on an analysis of literature (Pearson et al., 2015) and personal accounts (Owens et al., 2018), we set out to learn from services that were providing support and/or treatment for people with mental health problems who are involved with the CJS and where there was little or no published literature.

## Method

### Design

We used a multiple case study design (Yin, 2003), with a focused ethnographic approach (Knoblauch, 2005) as we wanted to understand the complex phenomena within their contexts and explore differences within and between cases.

### Selection of cases for study

To select suitable cases, we developed a set of criteria based on components of our emerging ‘Engager’ prototype intervention, particularly those areas where we had identified gaps in our current knowledge or needed better understanding (Table 1).

**Table 1:** Criteria for case study selection

1.	Service delivers psychological therapy to justice involved groups.
2.	Service delivers through-the-gate working.
3.	Service has established and developed care pathways linking different agencies both within and outside of the prison.
4.	Service has established and developed joint care plans/formulations between health and criminal justice agencies.
5.	Service is involving peers to offer/deliver support.
6.	Service is preparing and supporting people coming to the end of an intervention/therapy.

Services with potential to shed light on those areas were identified through a call to all Offender Health Research Network members, a broad internet search, and targeted examination of relevant criminal justice and health related websites. Thirty services were identified, details of which were used to populate a grid based on the criteria in Table 1. All 30 services met at least one criterion, but those only meeting one were not taken forward as we were also interested in how these aspects of services might influence one another. The remaining cases which met more than one criterion were reviewed by the research team and those finally selected were services felt by the research team to be most important for our developing theory and which were also willing to participate in the research.

Four case studies were selected and analysed. Service names have been removed to maintain anonymity. Table 2 maps each case study onto our six criteria.

**Table 2:** Case studies selected against questions

Questions to be answered from case studies						
	Psychological Therapy	Through-the-gate	Care pathways linking agencies both within and outside the prison	Joint plans/formulation between health and criminal justice agencies	Peer involvement in support/delivery	Preparations for ending support
Case Study						
1		✓	✓		✓	
2	✓		✓	✓		✓
3	✓	✓	✓	✓		✓
4	✓					✓

Case study 1 was a large male prison with a dedicated resettlement wing with well-developed through-the-gate services. The prison had a range of health and social care services working in close collaboration with the prison, developing shared care pathways and joint planning. In addition, it had a well-established model of peer support.

Case study 2 was a probation service which also had a co-located IAPT service (psychological therapy) within the same building. Interagency agreements between probation and the health service delivering the IAPT service had been established at the beginning of the co-location of the services and they had been trying to implement more joint working. In addition, the IAPT service supported clients in thinking about how to plan for the end of the psychological therapy.

Case study 3 was a service that was jointly funded by Her Majesty’s Prison and Probation Service (HMPPS) and the local NHS trust with clear joint working arrangements. The service provided a range of therapeutic approaches and psychological therapy for offenders with personality disorders, on licence under probation supervision. Clients were identified in prison and supported through-the-gate into the community, with sustained support while under licence and fully developed preparations for completion of the intensive intervention.

Case study 4 was a community service providing support for young people aged 16–22 who had been experiencing severe emotional distress. The service provided a range of therapeutic approaches and psychological therapy (including mentalisation). In comparison to case study 3, which was a long-term intensive intervention, this was a time-limited approach and therefore prepared young people for the ending of support, but over a much shorter period.

### Data collection

**Fig. 2:**
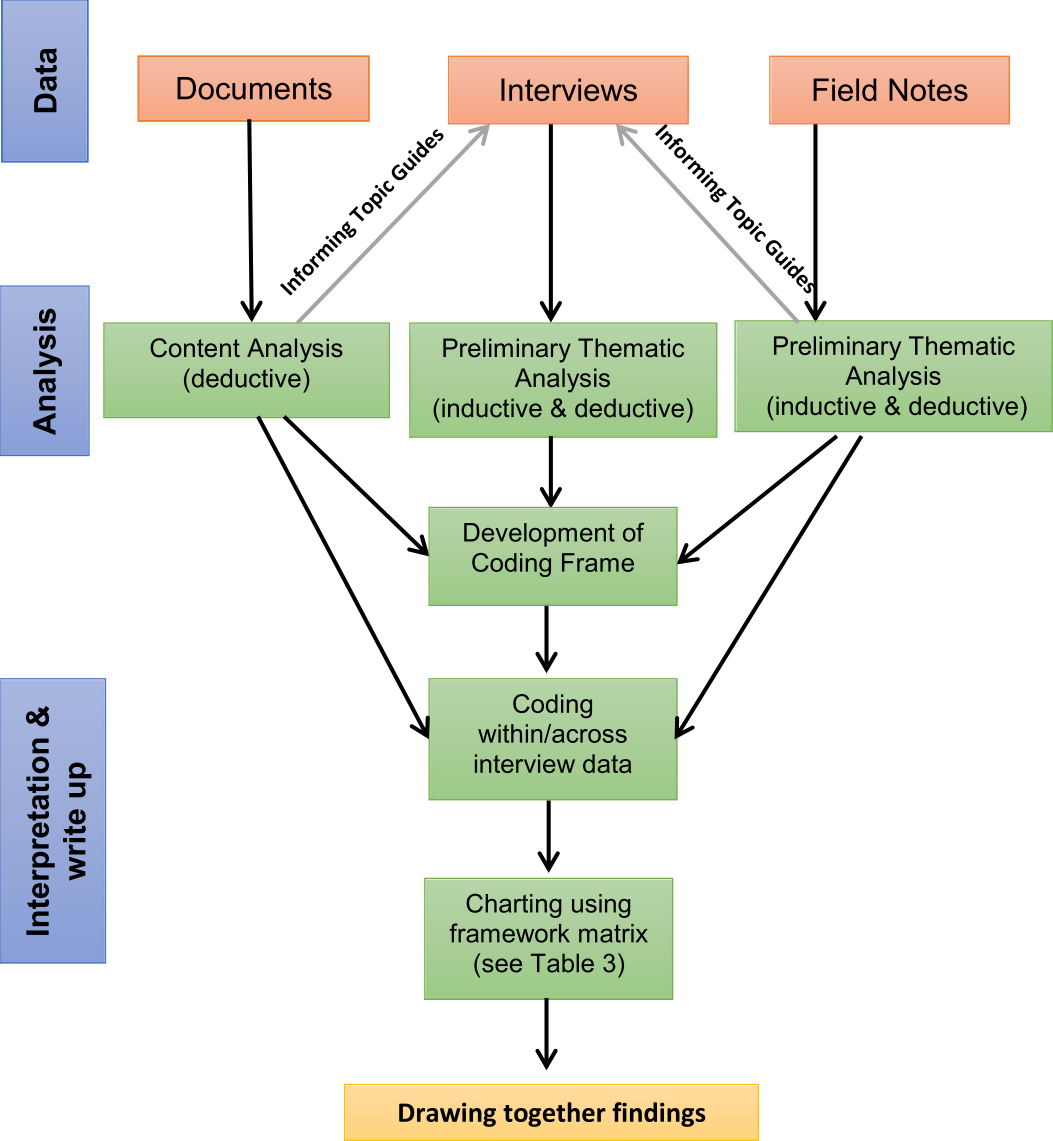
Data collection and analysis

For each case study, data came from three sources, namely documents, field notes and semi-structured interviews (See Fig. 2). Relevant documents (for example, internal and external evaluations, reports and audits, service information aimed at service recipients, standard operating procedures) relating to each case study were collected in advance of a visit. All documents were read and underwent content analysis. Examining the printed and published data in this way, before the visit, allowed us to identify questions and areas about which we wanted more detail.

Ethnographic data collection occurred between January 2014 and February 2015. A researcher [CL or SB] spent 2–4 days at each case study site, observing the service and the way it functioned. They made field notes, clearly distinguishing between what they actually observed and their own thoughts about their observations. They also conducted semi-structured face-to-face interviews with managers, practitioners and service recipients (10–15 informants at each site). Interviews focused on how the service did and did not work, for whom, and how outcomes could be improved. Realist interview schedules were constructed using the context-mechanism-outcome (CMO) configurations in the initial program theory (see Brand et al., 2019, for more details on how realist methods were used to develop the program theory). The developing program theory informed iterative revision of interview schedules.

All participants were provided with an information sheet explaining the details of the study, including information on how to withdraw and how data would be anonymised and remain confidential. All interviews were recorded on an encrypted Dictaphone. Interviews were transcribed as soon as possible after the interview and anonymised at the point of transcription. The audio file was then securely deleted.

### Analysis

Figure 2 provides an overview of data collection and analysis. All documents were analysed using content analysis and taking a deductive approach. Interviews were audio-recorded and transcribed verbatim. A preliminary thematic analysis (inductive & deductive) was undertaken of the interviews and field notes. A framework approach (Ritchie & Spencer, 1994) was adopted, following the five steps described below.

#### Familiarisation

Two researchers (CL & RS) immersed themselves in the raw data by listening to recordings, reading and re-reading all transcripts and documents, and making notes or analytic memos on spontaneously arising topics of interest, key ideas and recurrent themes.

#### Developing a coding framework

A coding framework was developed iteratively, using spontaneously arising topics of interest, issues raised by the participants and views or experiences that recurred in the data (inductive codes), as well as a priori (deductive) codes derived from the CMOs of initial program theory.

#### Coding

The coding framework was systematically applied to all the data, using NVivo 11 software to assign sections of text to particular codes. Transcripts were coded by CL or RS and 20% of transcripts were double coded and checked for consistency by CL. Any disagreements were resolved by discussion and arriving at a consensus with the wider team. The end product was a detailed index, which sorted the data into labelled chunks for further exploration.

#### Charting

Indexed data were entered into a framework matrix containing separate rows for each theme and columns for each case. The cells were populated with summarised data, including direct quotes from documents and interview participants, and reflective field notes. The completed matrix provided a visual aid to organisation, analysis and interpretation, facilitating identification of patterns within the data.

#### Mapping and interpretation

During this stage we pulled the key characteristics of the data from each case study, identifying cross-cutting themes that worked across all four case studies (Ritchie & Spencer, 1994). Two researchers (CL & RS) took on the main responsibility for undertaking the initial interpretation and mapping of the data, with the rest of the team being used as a sounding board, to check the persuasiveness of the analysis. The cross-cutting themes were expressed as narrative summaries, of the evidence for each component of the intervention. The results of this stage informed a stakeholder consensus group meeting and fed into decisions about which components of the ‘Engager’ intervention should be prioritised, amended or discarded in the main trial.

### Ethical approval

Ethical approval for the study was given by the East of England – Essex Research Ethics Committee (reference number 13/EE/0249). National Offender Management research approval was given in October 2013 (reference number 2013–187). Local research ethics (Research & Development) approval and the appropriate site-specific assessments were obtained from the relevant NHS Trusts at each case study site.

## Results

The seven themes and seven sub-themes identified, as shown in Table 3, are reported in the results below. How these findings were then incorporated into the ‘Engager’ complex intervention is detailed in the ‘Discussion’ section.

**Table 3:** Condensed framework matrix showing themes, sub-themes and data included from each case study

Theme	Sub-theme	Case Study			
		1	2	3	4
Collaboration (inter-professional practice)		✓	✓	✓	✓
	Shared information	✓	✓	✓	✓
	The whole is greater than the sum of its parts		✓	✓	
Client engagement		✓	✓	✓	✓
	Clear communication	✓	✓	✓	✓
	Building Rapport and Trust	✓	✓	✓	✓
	Time		✓	✓	✓
Client motivation			✓	✓	✓
	Timing		✓	✓	✓
	Preparation		✓	✓	✓
Supervision				✓	✓
Therapeutic style/approach		✓	✓	✓	✓
Peers		✓		✓	
Preparations for ending support			✓	✓	✓

### Collaboration

In all four cases, staff interviewees highlighted the importance of collaboration within and between multidisciplinary teams. Often this consisted of individuals who were motivated and supported by their own service to work within and across service boundaries. The importance of shared practice(s) was discussed by many and this was vital in enabling collaboration when working with complex groups.

In two of the four cases (Case Studies 2 & 3), health and criminal justice agencies were in the same building. Shared working practices between the two had been established from the very beginning, with both agencies being fully on board with the set-up of the service, with clear joint goals and responsibilities. This inter-professional working was at all staffing levels, from commissioners and senior management to front-line staff. During the setting up of the service, regular joint meetings were held in which the teams “*bashed out all the issues*” (Case Study 2; Staff Participant 1) and worked to reach an agreed set of goals and practices. Achieving such good relationships between health and criminal justice agencies took time and required hard work from both partner agencies, and the relationship required continuous fostering. For example, in Case Study 2 understanding between the two agencies was sustained by drop-in surgeries, at which professionals could answer each other’s questions and clarify processes and criteria for referral to their service. In case study 3 joint forums were actively encouraged where the health and justice teams could meet as a whole team: “*… in the [whole] team we will be able to chip in and it just feels fairer and feels more empowering and more flexible”* (Case Study 3; Staff Participant 3).

Staff participants reflected on previous roles in other organisations that did not have such well-established and embedded collaboration from service inception: where this happened, services were more likely to exist in silos. A participant said:

*“what the prison thinks is important for the resettlement wing, what prisoners think is a resettlement wing, and what agencies that want to work with prisoners think is important, all those three things, you need somebody to oversee it and coordinate it, and there actually isn’t any coordination of services at [prison]. But we all coordinate how? Who we’re working with and how we work together, I think that’s probably the bit that’s missing.”* (Case Study 1; Staff Participant 4)*.*

#### Shared information

In the two cases that had shared practices (Case Studies 2 & 3), they were established at the cross-organisational level and were seen to support information flow and make information sharing quicker. Furthermore, fast and open communication between health and criminal justice staff meant that clients’ needs could be responded to more appropriately and positive outcomes were more likely, for example, using knowledge from both services to better understand why clients may have missed appointments, or managing risk collaboratively. One staff participant said:

*“I think sharing information has definitely changed my practice. The ability to take a calculated risk and work together and recognise that this is a part of somebody’s, behavioural difficulties, traits and try and work with that and maybe not just recall them without exploring other ways of working”* (Case Study 3; Staff Participant 3).

An additional benefit of this improved sharing of information was that it created learning opportunities, with health and criminal justice staff being able to increase their knowledge about the other service and about client-related issues more generally. Better information flow between services was also noted to save resources, ensuring that work was not duplicated. It ensured that a consistent and cohesive response across both agencies was provided to clients and this reduced the risk of miscommunication between services.

In cases without this level of collaboration (Case Studies 1 & 4) there appeared to be inflexible and organisation-specific bureaucracy, which impeded collaboration and meant that clients had to provide the same details repeatedly. One staff interviewee commented that:

*“Everyone’s like ‘No, we have to use our own form’. Maybe if we had some kind of case management system where we could all input, you know, a section so that you could go on and you would know exactly what was happening with everybody and how it was all going to fit together”* (Case Study 1; Staff Participant 4).

#### The whole is greater than the sum of its parts

In the two cases with well-established collaboration between health and criminal justice agencies (Case Studies 2 & 3), staff suggested that their ability to manage complex groups was better as a whole than as individual services or practitioners:

*“it’s relying on the expertise of the team, we’ve all got different skills and we’ve come from different backgrounds and we’ve all got different opinions, so it works well … with the best will in the world, I can’t do everything”* (Case Study 3; Staff Participant 3).

In this case they described their approach as being like ‘*magpies’:*

*“The needs of the men means we have to make use of all the resources within the team, we’re like magpies, there isn’t a group that hasn’t been done at least once before, we’d identify a need for that and then we’d all work together to pull the programme together to meet those needs and then support staff to deliver that”.* (Case Study 3; Staff Participant 1).

Maintaining the effectiveness of this balance between individual professionals and a whole team approach required time and effort from the whole service. Being clear about roles and responsibilities ensured that that there was no duplication and that one agency did not undermine that work of another, *“it’s all about how you can complement each other, not duplicating what someone else is doing”* (Case study 2; Staff Participant 3).

### Client engagement

In all four case studies, client and staff interviewees talked about what were the key components of establishing and maintaining client engagement. These were: clear communication, building rapport and trust, and time.

#### Clear communication

Clear, open and honest communication was discussed by interviewees (staff and clients) across all case studies in relation to generating trust and maintaining engagement by managing client expectations. From the outset, a positive relationship was more likely to develop if clients felt that they were being properly informed. However, as one staff participant stated, many clients had not experienced this clear, open and honest communication before:

“*Well the first thing is not to fob them off. These people have been used to being fobbed off and failed, so I, I always say to them at the outset that I won’t promise them anything that I can’t deliver … and that doesn’t always make me very popular but at least people know that that’s where they stand. This group is very vulnerable, and they’ve had that happen a lot, and they expect it, they’re quite surprised when people actually follow through on what they say, which I think’s a shame*” (Case Study 1; Participant 2).

It was suggested that adapting one’s style of communication for each client was useful in maintaining engagement and that this might also change across the course of the relationship. Several staff described using alternative methods of communicating with their clients, aside from traditional talking therapy and work sheets, which could mean they became overwhelmed and demotivated. One staff participant described creating a collage with their client, as this moved the focus of attention from them and allowed them to express themselves in a non-verbal manner. Other staff suggested engaging in activities such as walking, visiting farms or other such social activities in order to strengthen their relationship with clients, but more importantly ensuring that conversations were in a more ‘naturalistic’, less formal and clinical setting.

It was noted by staff that professionals should be both positive and empowering in their communication with clients:

“*There’s something important about being clear about celebrating and noting success that really helps support the relationship*” (Case Study 3; Staff Participant 1).

Another said that:

“*I like to be as open as possible with the client, to say this is your session, you’re a partner with me [ … …*] *you can help me guide the session*” (Case study 2; Staff Participant 1).

It was further highlighted that honesty and clarity of communication should also extend to staff openly admitting when they have made mistakes to demonstrate respect to clients.

#### Building rapport and trust

A commonality across all four case studies was the view that clients had in the past experienced very difficult personal relationships and at times very difficult relationships with professionals. As a result, building trust and rapport, while noted as sometimes difficult, was vital for maintaining engagement; “*I certainly think that’s what brings them back, it is because I am who I am. It is about relationships*” (Case study 4; Staff Participant 1a). The benefits of developing trust and rapport with staff were clear to see from these clients’ comments:

“*I opened up more than I have opened up to my own family … she just puts you at ease*” (Case study 2; Client Participant 8).

“*I don’t see them as key workers, now I see them as friends, you just develop that relationship where you can speak freely but that wasn’t the case at the beginning*” (Case Study 3; Client Participant 7)

Continuity was important in developing trust and rapport:

“*They [clients] find it difficult to trust people, keep it simple and have one person that they deal with, you know, throughout. It helps them build that rapport with the person … I don’t think people realise what a massive impact that has on somebody, having a different worker*” (Case study 2; Staff Participant 9).

Highlighted in all case studies was the importance of staff showing respect to clients; this was the foundation for developing trust and rapport and was crucial for their continued engagement. As one client reported: “*I’ve only seen respect and courtesy from [staff at this service], you know what I mean, so, and most of the lads are quite brought round by it”* (Case Study 1; Client Participant 5).

#### Time

The concept of time was an important factor in engaging clients. Having time was necessary to build trusting and supportive relationships to sustain engagement in therapy, especially amongst complex clients. It was generally agreed that the longer a relationship had been in existence, the more robust it was. For that reason, relationship building should begin as early as possible, and if possible, with work taking place well before any significant transitions e.g. between prison and the community.

The development of therapeutic relationships over time was regarded as important not just for transitional phases, but also for the client to feel comfortable and supported enough to share their life experiences with professionals. For example, in Case Study 4 they had a 12-week assessment period which was regarded as beneficial for engagement because the clients didn’t have to tell everything in an hour but could build trust over time. Additionally, it was argued, the more time spent with a client, the more understanding professionals they will have of them, allowing the staff member to provide better care.

### Client motivation

The case studies identified that, once a client had engaged with services, it was important to understand their motivations to continue, how soon to start more formal therapy and when to expect behaviour change.

#### Timing

Many client participants believed there was a ‘right time’ at which people were more willing to engage with therapy and to change their behaviour. One client reported that the right motivations were key, arguing that: “*you’ve got to be in the right frame of mind, you’ve got to want to take part*” in therapy for it to be successful (Case Study 3; Client Participant 7). Another discussed how, as a younger man, he would not have engaged with some aspects of the therapy that he now found helpful:

*“If you’d have said anything to me about doing meditation back in 1995, I’d have said, what, you having a laugh or something? It would never have happened … you know what I mean. First because I wasn’t into that sort of thing, and second because I didn’t have the confidence. My confidence and self-esteem were so low that it was unbelievable. There was no way you would have got me in here with a bunch of people doing all sorts of weird movements and stuff. It just wouldn’t have happened. But that’s another way in which I’ve changed. And I am more open to these sort of things”* (Case Study 3; Client Participant 9).

#### Preparation

Linked to this was the view that there was a ‘right time’ to begin more formal therapy and that this could be created by careful preparatory work. This consisted developing ‘emotional literacy’, so that the client felt more comfortable naming emotions:

*“I think it’s really important for people to have a narrative. The emotional literacy provides a narrative, a way of articulating what’s going on … I can have a conversation with somebody very quickly because we have established a context for particular words to describe what it is they’re feeling”* (Case Study 4; Staff Participant 2b).

In case study 2, criminal justice staff played a vital role in explaining what therapy would be like, preparing them to start thinking about their thoughts and behaviours and managing their expectations, as many had had negative experiences with mental health services in the past.

*“I think it’s about how you explain things to people, because some people think they’re going to talk about their childhood abuse and they’re going to talk about really deep-rooted issue. I don’t explain it like that, I say, your past abuse isn’t going to suddenly disappear, it’s about how you can deal with that. That’s about preparing for therapy, if you don’t do that with them then they’re obviously going to have unrealistic expectations”* (Case Study 2; Staff Participant 9).

### Supervision

In three out of the four cases (Case Studies 2, 3 & 4), supervision was mentioned as a vital aspect to support staff when working with complex clients. Supervision could ensure that staff were not left feeling “*unheard or neglected”* (Case Study 3; Staff Participant 1). One staff participant suggested: “*I think it’s helping the staff members feel a little bit more contained, hopefully a bit more guided, confident about what they’re doing in their work really”* (Case Study 3; Staff Participant 6). Another simply that supervision *“keeps us safe and keeps us sane”* (Case study 4; Staff Participant 1.2a).

Different types of supervision were seen to have different benefits. For example, the necessity for a *“managerial eye”* to be kept on the work with clients was summed up by this participant:

*“We’re asking people to do quite complicated work really and if they haven’t got someone to check in a detailed way about how that work’s going, then it drifts”* (Case study 3; Staff Participant 6).

However, supervision as a form of emotional support was thought to be more important:

*“We recognise here that the client can be very damaging to us, in terms of just feeling quite overwhelmed, just feeling that they can trigger a lot of your own kind of schemas, um, and it’s important that people have a place to talk about that”* (participant 3.6)

Supervision, as a form of emotional support, was not only crucial for the well-being of staff, but also enabled them to work more effectively with other professionals and clients. As one staff interviewee stated:

“*colleagues will work better with each other if they are given space to vent their feelings*” (Case Study 3; Staff Participant 1).

The professional who provided the supervision was also important. In two of the four case study sites, there had been a shift from a professional internal to the service to an external provider and vice versa. Both cases highlighted that, from their perspective, a professional external to the service was best and improved the quality:

*“It all got a bit messy in terms of roles [when we had internal supervision] and so we decided to get an external supervisor. On the whole, that is better”* (Case study 3; Participant 3.1).

### Therapeutic style/approach

All four case studies included some discussion on therapeutic style or approach. Across the case studies there was a wide variety of different therapeutic approaches, including: counselling, Cognitive Behavioural Therapy, Psychotherapy, Cognitive Analytic Therapy, Transactional Analysis and Mentalisation. Services used a range of techniques, as it was not possible to “*shove the client into a model*” (Case Study 2; Participant 1). Intervention type very much depended upon the assessment of the individual’s needs and goals and needed a very pragmatic approach.

*“We have to be pragmatic, we had all these high aims of getting people into paid employment and lots of ideas about how to do that. And then we realised that a lot of these men struggled to sit in a room with other people in a non-conflictual way. We’ve had to moderate our expectations and approach, but still with an optimistic stance and not giving up on people*.” (Case Study 3; Participant 1).

Mentalisation-based approaches were used in two of the four case studies (Case studies 3 & 4). On one case to *“help [clients] be more effective in their relationships, it helps then to keep another person’s mind in mind*” (Case Study 3; Participant 6). For staff, “*it’s part of the infrastructure of their work and is used all the time, particularly for recognising what’s in our minds isn’t necessarily apparent to the person we’re working with*” Case Study 3; Participant 1). While both case studies used Mentalisation, it was an approach rather than Mentalisation-based therapy; “*I think it can be perceived and is often delivered in quite a complicated way … we’ve just kind of shortened it … kind of changed it to be more usable*” (Case Study 3; Participant 6).

The same professional noted that they also used elements of Cognitive Analytic Therapy as part of this approach:

“W*hen the men leave, key workers do write letters, and all the staff are invited to write a little paragraph to the men about reflecting on their time here, how the key workers have experienced them, what they’ve noticed about their lives, that’s kind of resonated with them and some of the areas to work on. I just think it’s a really powerful way, it helps the men to feel quite contained and held and understood*” (Case Study 3; Participant 6)

Modelling positive relationships was also seen as an important approach used in Case Studies 3 & 4 “*your relationship with them is a way of them learning what it’s like to be in a positive relationship*” (Case Study 4; Participant 2b). This might involve actually role-playing scenarios: “*I give them absolute permission to use me as a guinea pig for the way they might want to learn about being in a relationship*” (Case Study 4; Participant 2b).

### Peers

One of the main reasons for selecting Case Study 1 was the service’s use of peers within the prison setting as ‘healthcare reps’. Healthcare Representatives support prisoners’ access to health services and undertake a range of duties, including: distribution of appointment slips; reminding other prisoners of appointments; acting as listeners and advocates for health and social care issues; attending regular meetings with healthcare staff to share service updates and feedback from other prisoners and, providing healthcare information to new prisoners. All professionals interviewed in Case Study 1 felt that the use of peers as healthcare representatives was an excellent initiative. Observations from professionals suggested that it helped the peer by increasing self-esteem, self-advocacy and a sense of responsibility. As well as direct benefits to the peer, healthcare professionals felt that the healthcare representatives had improved services within the prison and acted to improve communication between other prisoners and healthcare:

“*healthcare reps (peers) are pretty good at being that in-between, which is important cos sometimes lads don’t want to say anything, they struggle forever, and it may well be that they will come and tell you something, do you think he’ll talk to me. It’s that, it’s that bridge between sort of them and us really*” (Case Study 1; Participant 2).

However, one professional felt that the healthcare reps initiative only worked because it had a dedicated, full-time manager and without that it would have failed. Also they highlighted that there had been several incidents where the healthcare rep position had been abused and therefore any peer involvement needed robust procedures.

### Preparations for ending support

Participants at three of the four sites talked about how they prepared their clients for the time when the therapy or service came to an end (Case Studies 2, 3 & 4). In all three cases, professionals talked about the importance of planning for the end well in advance and reminding and counting down the number of sessions left. Professionals also highlighted the importance of framing the end, not as finite, but as only part of the person’s journey. One professional stated:

“*So they’ll often come in and say, what’s the point in talking to you, you’re just going to up and leave after two years, aren’t you? I say, then maybe I can occupy one chapter in your book and let’s see what we can do and how we change your story.”* (Case Study 4; Participant 2b).

## Discussion

The aim of the case studies was to fill the gaps within our knowledge and provide practical insights for the development of the ‘Engager’ program theory and practice. This was achieved by identifying key cross-cutting themes of our perceptions of effective practice within health and justice services and how these practices worked to engage and retain clients. We were able to examine how these themes worked in combination, as well as singularly and explore similarities and differences to how they functioned across a variety of cases. This new knowledge was incorporated, along with learning from the other parts of the ‘Engager’ programme (Brand et al., 2019; Lennox et al., 2017; Owens et al., 2018; Pearson et al., 2015) into the four key elements of the Implementation Delivery Platform: the manual, the training, supervision and interagency organisational agreements.

### How the case studies changed the ‘Engager’ implementation delivery platform

The findings from the case studies were critical to the development of the ‘Engager’ intervention manual, by providing practical guidance for practitioners about how to achieve, for example engagement and trust with clients and effective collaboration with other services. The original model for the psychological therapy component within ‘Engager’ was based on an IAPT style approach, but all case studies highlighted that this alone would not meet the complex needs of the client group. They highlighted the clear need for an intervention to have in its toolbox a range of psychological therapies and that the addition of a mentalisation-based approach would be beneficial to support the relationship between the practitioner and client. The case studies also led us to focus specifically on the role of supervision for the ‘Engager’ team, and the additional need for external supervisors. As a result, each ‘Engager’ team consisted of at least two practitioners and a supervisor who provided day-to-day case management and internal supervision, with a senior clinical psychologist with expertise in mentalisation-based approaches providing external supervision. The case studies altered our understanding of when the intervention should begin and how it should end. Within the pilot trial (Brand et al., 2019; Lennox et al., 2017) the intervention delivery began 12 weeks pre-release. The case studies highlighted the need for time and significant amounts of preparatory work to build trust and rapport. Therefore, we increased the time available for delivery of the intervention within the prison from 12 to 16 weeks. In addition, the case studies demonstrated the need to prepare clients for the end of an intervention, starting at the commencement of the intervention and talking about how they would like their ending to be. An important part of this was framing the ending as a positive step in a longer journey and the case studies suggested providing clients with a summary of their progress. Finally, one aspect that was in the initial ‘Engager’ prototype was the use of ‘Engager’ peers to support engagement and improve outcomes. Evidence from the case studies suggested clear benefits for peer involvement, but less benefit for the recipient and that involving peers in interventions required significant planning and management to avoid adverse incidents and abuse of responsibilities. A systematic review of the effectiveness of peer education and peer support in prison echoed the benefits for the peers but less robust evidence for benefits for recipients (Bagnall et al., 2015). Therefore, based on this evidence the team decided not to include ‘Engager’ peer support, but if the individual wanted peer support/mentor the ‘Engager’ practitioner would encourage and facilitate accessing a peer/mentoring service.

### Key elements of interagency working

These case studies highlight that engaging and motivating clients is dependent on the relationship built with the professional, which is developed through building trust and rapport. This in turn, required time and respectful, open and honest communication. The case studies also highlight that professionals are often unable to provide this effectively if they themselves did not work in effective interagency collaborations that were greater than the sum of their parts, had shared practices and effective supervision. Without these, professionals often reverted to silo-working and restrictive practices, which impacted directly on their relationships with clients and in turn on clients’ outcomes. In addition, not taking a ‘one size fits all’ approach to therapeutic style was important, given the complexity of the client groups, but this relied on having a very skilled and well supervised workforce to be able to deliver combined approaches.

Numerous studies have identified the relationship or therapeutic relationship as being essential to the creation of positive client experiences (Lambert & Barley, 2001). Personal qualities such as empathy and warmth are more associated with positive client outcomes than any specific specialised treatment intervention, and some studies have suggested that the relationship is by far the most important factor (Stamoulos et al., 2016). However, what is interesting in these case studies is that professionals show how client relationships can be damaged or adversely affected by dysfunctional interagency collaboration.

It was clear from these four case studies that health and justice services sharing common practices was vital to the development of relationships. The impact of shared goals on collaboration has been discussed frequently throughout the literature (Harris, 2003), as has the impact of good communication, and willingness to share information (Sloper, 2004). It is often assumed that interagency working based on shared knowledge and expertise will be effective (Nash, 2006), but it has been argued that when working with complex client groups, such as those involved with the criminal justice system, this needs to be more formalised with clarity on roles and responsibilities and clear accountability (Watson, 2010).

Two of our case studies highlighted that the management of complex client groups was better done by services working collaboratively together rather than as individual services or practitioners. However, maintaining a balance between wider team membership and retaining individual professional identity can be difficult. This is made more complex where the boundaries between individual job roles and roles in the collaborative process become blurred. Rose and Norwich (2014) define this as ‘identity dilemma’ whereby there is a conflict between deep, but bounded, specialist knowledge and wider knowledge which spans professional boundaries. They argue that it can take time to develop a clear idea of what it means to be a hybrid or ‘interagency’ professional in practice, which can result in practitioners feeling insecure within such a role. Evidence from these two case studies suggests that those involved overcame these dilemmas by putting in time and effort at the beginning of setting up the services with clear roles and responsibilities for interagency professionals and the regular forums.

The case studies highlight many of the same issues encountered in inter-professional working with complex groups. Hood (2012) presents a critical realist model of complexity for considering these issues. He argues that it is ‘naïve’ to disaggregate complex needs into individual needs that are then targeted by specific interventions and professionals, with pre-specified outcomes. Needs are complex, and each need will have a range of other conditions that exert an influence, for example individual characteristics, poverty or deprivation. Furthermore, of all the possible events that could take place, only certain ones will be recorded as the outcome for the particular intervention. In addition, Hood describes the process of ‘reflexive-hermeneutic complexity’. For professionals, applying their expertise is a social and individual process, which will be shaped by a range of factors, including what they have learned, what they are mandated to do, and by their interactions with clients and other professionals. Hood suggests that a way of managing complexity would be inter-professional practice characterised by coordination and information sharing, an arrangement between agencies and practitioners to agree clear goals and responsibilities and open and honest communication. However, that can increase complexity further, as each member brings new knowledge and their own understanding. Inter-professional collaborations need “double reflexivity”. They must integrate all the various pieces of expertise, experience and knowledge available to deal with the case in hand, while also managing the social processes entailed in doing so. Complexity means that the dynamics will change on an individual basis, requiring a kind of reflexive adaptability that goes beyond merely protocols and guidelines. This could involve some form of clinical or group supervision, which allows scope for creative disagreement as well as for consensus on more routine aspects. Through this supervision, practitioners may gain a better understanding of causal mechanisms than they could on their own. The case studies presented here highlight elements of this reflexive adaptability: in two of them, clinical and group supervision was used as a ‘safe space’ for health and justice professionals to come together and key to this for both services was the use of a professional external to the service.

The strength of this study is the use of a multiple case study design as it allowed us to extend beyond a descriptive and explorative case study design, enabling greater rigour in research (Fisher & Ziviani, 2004). Multiple case study design can create a more convincing theory where the evidence comes from more than one source (Eisenhardt & Graebner, 2007). A particular strength was the decision to focus on novel and forward-thinking services where there was limited robust evidence-based literature to inform delivery.

However, there are also a number of limitations. The main driver for the selection of cases was trying to fill our knowledge gaps in our developing program theory and Intervention Delivery Platform. Therefore, the selection was biased towards specific health and justice services delivering elements of our emerging program theory. By their very nature the cases were not necessarily representative of or generalisable to all health and justice services. Another limitation is the small number of cases studies. Six case studies were originally planned for. However, we experienced delays in obtaining approval for two. As this was part of a larger study, we needed to conduct all case studies within a particular time period due to the development of the program theory being used to develop the intervention that would be tested within an empirical trial. Therefore, a decision was made by the research team not to pursue them. It is possible that these two case studies may have altered our program theory or given us new insight into interagency working. While multiple case studies are likely to more representative than a single case, case studies are expensive and time-consuming to undertake. Therefore, the more cases undertaken the more likely it is that less data is collected for each case (Baxter & Jack, 2008). In this study we collected documents that were provided from the service. It is possible that they may have withheld some documents if they felt that they were sensitive or may have shown them in a negative light. We have no evidence to suggest that this happened, but it is a possibility. The researcher spent 2–4 days at each case study site, observing the service and the way it functioned. It is possible that on the days the researcher was there the service functioned differently due to their presence. Also, we conducted interviews only with 10–15 informants at each case study site. The recruitment of informants was designed to be open and transparent and to minimise bias: for example, an email was sent to staff and clients with information about the study and the researcher’s contact details and anyone who wanted to take part was asked to contact the researcher directly. However, it is possible that the service took steps to control who this information went to. Again, we have no evidence that this was the case but is a possibility. All these factors may have biased our findings in some way.

## Conclusion

Individuals with common mental health problems in contact with the CJS have diverse and complex needs, so effective interagency collaboration is essential to maximise outcomes and avoid individuals slipping through the net. However, effective interagency collaboration is also complex and often fails to deliver benefits (House of Commons, 2018; National Audit Office, 2017; Public Health England, 2016). Understanding this complexity is vital and ‘Engager’ recognised this by developing the invention using realist methods (Brand et al., 2019; Pearson et al., 2015). The case studies detailed here fed into the ‘Engager’ program theory by providing practical insights for practitioners and by emphasising important processes, such as how client relationships can be damaged or adversely affected by dysfunctional interagency collaboration. Such lessons come at a critical time, just when NHS England have engaged in a large-scale pilot of ‘through the gate’ intervention (RECONNECT) across England. By selecting collaborations that were innovative and forward thinking with a flexible approach, we could examine complex, multifactorial phenomena and practice models that played a valuable part in developing both the core practice and the Implementation Delivery Platform for an innovative complex intervention.

## Data Availability

Due to the population of research participants involved in this study individual-level data are not publicly available.

## References

[CR1] Bagnall, A., South, J., Hulme, C., Woodall, J., Vinall-Collier, K., Raine, G., … Wright, N. (2015). A systematic review of the effectiveness and cost-effectiveness of peer education and peer support in prisons. *BMC Public Health*, *15*, 290. 10.1186/s12889-015-1584-x PMC4404270 25880001

[CR2] Baxter P, Jack S (2008). Qualitative case study methodology: Study design and implementation for novice researchers. The Qualitative Report.

[CR3] Bradley K (2009). Review of people with mental health problems or learning disabilities in the criminal justice system.

[CR4] Brand SL, Quinn C, Pearson M, Lennox C, Owens C, Kirkpatrick T (2019). Building programme theory to develop more adaptable and scalable complex interventions: Realist formative process evaluation prior to full trial. Evaluation.

[CR5] Brooke D, Taylor C, Gunn J, Maden A (1996). Point prevalence of mental disorder in unconvicted male prisoners in England and Wales. British Medical Journal.

[CR6] Brugha T, Singleton N, Meltzer H, Bebbington P, Farrell M, Jenkins R (2005). Psychosis in the community and in prisons: A report from the British National Survey of psychiatric morbidity. American Journal of Psychiatry.

[CR7] Byng R, Quinn C, Sheaff R, Samele C, Duggan S, Harrison D (2012). Care for offenders: Continuity of access. NIHR service delivery and organisation programme SDO project.

[CR8] Department of Health (2009). Improving health: Supporting justice: The national delivery plan of the health and criminal justice programme board.

[CR9] Eisenhardt KM, Graebner ME (2007). Theory building from cases: Opportunities and challenges. The Academy of Management Journal.

[CR10] Fazel S, Danesh J (2002). Serious mental disorder in 23000 prisoners: A systematic review of 62 surveys. Lancet.

[CR11] Fisher I, Ziviani J (2004). Explanatory case studies: Implications and applications for clinical research. Australian Occupational Therapy Journal.

[CR12] Hamilton L, Belenko S (2016). Effects of pre-release services on access to behavioral health treatment after release from prison. Justice Quarterly.

[CR13] Harding, C., Wildgoose, E., Sheeran, A., Beckley, G., & Regan, E. (2007). *Study to undertake a mental health needs assessment across Kent and Medway prison estate*. Maidstone: Kent and Medway NHS and Social Care Partnership Trust.

[CR14] Harris S (2003). Inter-agency practice and professional collaboration: The case of drug education and prevention. Journal of Education Policy.

[CR15] Harty M, Tighe J, Leese M, Parrott J, Thornicroft G (2003). Inverse care for mentally ill prisoners: Unmet needs in forensic mental health services. The Journal of Forensic Psychiatry and Psychology.

[CR16] Hood R (2012). A critical realist model of complexity for interpersonal working. Journal of Interprofessional Care.

[CR17] House of Commons (2018). Health and social care committee: Prison health. Twelfth report of session 2017–19.

[CR18] Kirkpatrick T, Lennox C, Taylor R, Anderson R, Maguire M, Haddad M (2018). Evaluation of a complex intervention (Engager) for prisoners with common mental health problems, near to and after release: Study protocol for a randomised controlled trial. British Medical Journal Open.

[CR19] Knoblauch H (2005). Focused ethnography. Forum Qualitative Social Research.

[CR20] Lambert MJ, Barley DE (2001). Research summary on the therapeutic relationship and psychotherapy outcome. Psychotherapy: Theory, Research, Practice, Training.

[CR21] Lennox C, Kirkpatrick T, Taylor RS, Todd R, Greenwood C, Haddad M (2017). Pilot randomised controlled trial of the ENGAGER collaborative care intervention for prisoners with common mental health problems, near to and after release. Pilot and Feasibility Studies.

[CR22] Lennox C, Mason J, McDonnell S, Shaw J, Senior J (2012). Information sharing between the National Health Service and the criminal justice system in the United Kingdom. Journal of Forensic Nursing.

[CR23] Lennox C, Senior J, King C, Hassan L, Clayton R, Thornicroft G (2012). The management of released prisoners with severe and enduring mental illness. Journal of Forensic Psychiatry and Psychology.

[CR24] Light M, Grant E, Hopkins K (2013). Gender differences in substance misuse and mental health amongst prisoners.

[CR25] Maguire M, Hucklesby A, Corcoran M (2016). Third tier in the supply chain? Voluntary agencies and the commissioning of criminal justice services. The voluntary sector and criminal justice.

[CR26] Mair G, May C (1997). Offenders on probation, Home Office Research study 167.

[CR27] Mallik-Kane K, Visher C (2008). Health and prisoner reentry: How physical, mental, and substance abuse conditions shape the process of reintegration.

[CR28] Ministry of Justice (2012). Estimating the prevalence of disability amongst prisoners: Results from the Surveying Prisoner Crime Reduction (SPCR) survey.

[CR29] Ministry of Justice (2013). Transforming rehabilitation: A strategy for reform.

[CR30] Nash M (2006). Public protection and the criminal justice process.

[CR31] National Audit Office (2016). Transforming rehabilitation.

[CR32] National Audit Office (2017). *Mental health in prisons*. London: Her Majesty’s Prison & Probation Service, NHS England and Public Health England.

[CR33] National Audit Office (2019). Transforming rehabilitation: Progress review.

[CR34] Owens C, Carter M, Shenton D, Byng R, Quinn C (2018). Engaging without exposing: Use of a fictional character to facilitate mental health talk in focus groups with men who have been subject to the criminal justice system. Qualitative Health Research.

[CR35] Pearson M, Brand S, Quinn C, Shaw J, Maguire M, Michie S (2015). Using realist review to inform intervention development: Methodological illustration and conceptual platform for collaborative care in offender mental health. Implementation Science.

[CR36] Prison Reform Trust (2018). Bromley briefing prison factfile.

[CR37] Public Health England (2014). Health and justice report: No health without justice, no justice without health.

[CR38] Public Health England (2016). Rapid review of evidence of the impact on health outcomes of NHS commissioned health services for people in secure and detained settings to inform future health interventions and prioritisation in England.

[CR39] Rekrut-Lapa T, Lapa A (2014). Health needs of detainees in police custody in England and Wales: Literature review. Journal of Forensic and Legal Medicine.

[CR40] Ritchie J, Spencer L, Bryman A, Burgess RG (1994). Qualitative data analysis for applied policy research. Analyzing qualitative data.

[CR41] Rose J, Norwich B (2014). Collective commitment and collective efficacy: A theoretical model for understanding the motivational dynamics of dilemma resolution in inter-professional work. Cambridge Journal of Education.

[CR42] Shaw, J., Conover, S., Herman, D., Jarrett, M., Leese, M., McCrone, P., et al. (2017). Critical time Intervention for Severely mentally ill Prisoners (CrISP): A randomised controlled trial. *Health Services and Delivery Research*, *5*(8). Southampton (UK): NIHR Journals Library. 28252895

[CR43] Singleton N, Meltzer N, Gatward R, Coid J, Deasy D (1998). Psychiatric morbidity among prisoners in England and Wales.

[CR44] Sirdifield C (2012). The prevalence of mental health disorders amongst offenders on probation: A literature review. Journal of Mental Health.

[CR45] Sloper P (2004). Facilitators and barriers for co-ordinated multi-agency services. Child: Care, Health and Development.

[CR46] Stamoulos C, Trepanier L, Bourkas S, Bradley S, Stelmaszczyk K, Schwartzman M, Drapeau M (2016). Psychologists’ perceptions of the importance of common factors in psychotherapy for successful treatment outcomes. Journal of Psychotherapy Integration.

[CR47] Stewart D (2008). The problems and needs of newly sentenced prisoners: Results from a national survey.

[CR48] Taylor C, Gill L, Gibson A, Byng R, Quinn C (2018). Engaging “seldom heard” groups in research and intervention development: Offender mental health. Health Expectations.

[CR49] Toi H, Mogro-Wilson C (2015). Discharge planning for offenders with co-occurring disorders: The role of collaboration, medication, and staff. Journal of Offender Rehabilitation.

[CR50] Wallace D, Fahmy C, Cotton L, Jimmons C, McKay R, Stoffer S, Syed S (2016). Examining the role of familial support during prison and after release on post-incarceration mental health. International Journal of Offender Therapy and Comparative Criminology.

[CR51] Watson A, Pycroft A, Gough D (2010). Sharing or shifting responsibility? The multi-agency approach to safeguarding children. Multi-agency working in criminal justice: Control and care in contemporary correctional practice.

[CR52] Yin RK (2003). Case study research: Design and methods.

